# Diabetes and cancer: glucose control impact on survival and tumor outcomes

**DOI:** 10.1007/s11154-026-10040-x

**Published:** 2026-05-04

**Authors:** Rosaria Maddalena Ruggeri, Erika Maria Grossrubatscher, Giulia Arrivi, Eleonora Ciocca, Alessia Filice, Bianca Golisano, Rossella Mazzilli, Natalie Prinzi, Annamaria Colao, Antongiulio Faggiano

**Affiliations:** 1 Department of Human Pathology of Adulthood and Childhood DETEV, Endocrinology, University of Messina, Messina, Italy; 2 Endocrine Unit, ASST Grande Ospedale Metropolitano Niguarda, Milan, Italy; 3 Department of Clinical and Molecular Medicine, Oncology Unit, Sapienza University of Rome, Sant’ Andrea Hospital, 00189 Rome, Italy; 4 Department of Experimental Medicine, Sapienza University of Rome, Rome, Italy; 5 Department of Clinical and Molecular Medicine, Endocrinology Unit, European Neuroendocrine Tumor Society (ENETS) Center of Excellence, Sapienza University of Rome, Sant’Andrea University Hospital, Rome, Italy; 6 Department of Clinical Medicine and Surgery, Endocrinology, Diabetology and Andrology Unit, Federico II University of Naples, Naples, Italy; 7 UNESCO Chair “Education for Health and Sustainable Development”, Federico II University, Naples, Italy

**Keywords:** Cancer, Diabetes, Antidiabetic drugs, Hyperglycemia, Dysglycemia, Continuous glucose monitoring, Survival, Prognosis

## Abstract

Diabetes mellitus (DM) and cancer are major global health challenges that increasingly coexist due to shared risk factors including aging, obesity, sedentary behavior, and chronic low-grade inflammation. Beyond being a common comorbidity, DM—particularly type 2 diabetes—has emerged as an important modifier of cancer risk, progression, treatment tolerance, and survival. Epidemiological studies consistently associate DM with a higher incidence of several malignancies, including pancreatic, liver, colorectal, breast, and endometrial cancers, as well as increased cancer-specific and overall mortality. The biological link between dysglycemia and cancer is complex and multifactorial. Chronic hyperglycemia, hyperinsulinemia, and insulin resistance promote tumor development and progression through altered cellular metabolism (Warburg effect), activation of insulin and insulin-like growth factor pathways, systemic inflammation, oxidative stress, immune dysfunction, and changes in the tumor microenvironment and gut microbiota. This review summarizes current evidence on the interplay between dysglycemia and cancer and explores how integrating continuous glucose monitoring (CGM)-based strategies into multidisciplinary oncology care may improve both metabolic and oncologic outcomes. A comprehensive search of online databases, including PubMed, ISI Web of Science, and Scopus, was conducted to identify studies assessing the impact of glycemic disturbances and glycemic control on cancer outcomes. Poor glycemic control and increased glucose variability are associated with worse oncologic outcomes, higher rates of treatment-related complications, reduced adherence to therapy, and diminished efficacy of chemotherapy, targeted agents, and immune checkpoint inhibitors. Severe hypoglycemia has also emerged as an independent predictor of poor prognosis. Although HbA1c has long been the cornerstone of glycemic assessment, it incompletely captures the dynamic glucose fluctuations commonly observed during cancer therapy. CGM provides a more comprehensive and clinically meaningful assessment of glycemic control, with the potential to reduce hypoglycemia, improve glycemic stability, and enhance tolerance and adherence to anticancer treatments. Current evidence indicates that diabetes and dysglycemia are key modifiers of cancer risk, progression, treatment tolerance, and survival. Optimizing glycemic control may therefore contribute to improved cancer outcomes. CGM represents a promising tool for personalizing diabetes management in oncology settings.

## Diabetes and cancer: an intriguing relationship

Diabetes mellitus (DM) and cancer are two of the most prevalent and burdensome chronic diseases worldwide. In recent decades, their coexistence in the same individuals has become increasingly common, raising important concerns for both clinical management and public health. This growing intersection is largely attributed to shared risk factors such as aging, obesity, sedentary lifestyle, poor dietary habits, and chronic low-grade inflammation [1–3]. As the global prevalence of diabetes continues to rise—with projections estimating over 780 million affected individuals by 2045—the number of patients living with both diabetes and cancer is expected to increase proportionally. Beyond their shared risk profile, there is growing recognition of diabetes as a potential modifier of cancer risk and outcomes.

The biological mechanisms underlying this association are complex and multifactorial. Chronic hyperglycemia, hyperinsulinemia, insulin resistance, and elevated levels of insulin-like growth factor-1 (IGF-1) are believed to play key roles in promoting cellular proliferation and inhibiting apoptosis, thereby fostering a tumorigenic environment [4, 5]. Furthermore, diabetes-induced alterations in the tumor microenvironment, immune dysfunction, and systemic inflammation may further contribute to cancer development and progression [6, 7]. In addition, type 2 diabetes (T2DM) is often associated with obesity, another condition linked to an increased risk of cancer, and the underlying pathophysiological mechanisms are likely to overlap and reinforce each other [8, 9].

Approximately 8–18% of oncologic patients have DM, and epidemiological studies suggest that DM, particularly T2DM, is associated with a 15% to 25% increased risk of developing cancer, although this risk varies substantially across different cancer types [10–13]. For example, an association between DM and pancreatic cancer has been clearly established [14], and DM is also associated with a 97% increased risk of intrahepatic cholangiocarcinoma and endometrial cancer [1, 15]. A recent observational study involving 44 million individuals reported an increased risk of breast and colorectal cancer and a reduced risk of prostate cancer [12]. These findings are consistent with a meta-analysis by Ling et al. [11], which included 151 cohort studies and over 32 million individuals. However, the relationship between chronic hyperglycemia, for which HbA1c is a commonly used biomarker, and cancer remains complex and not fully understood, and effects differ by cancer type, baseline risk, glycemic range, or duration of exposure. In a systematic review by Hope et al., higher HbA1c was correlated with increased risk of colorectal, pancreatic, lung, and female genital tract cancers, especially in individuals without diabetes, while no correlation was reported in breast cancer, gastrointestinal or urological neoplasms [16]. Conversely, this association was not confirmed in a large Swedish prospective cohort, suggesting that within diabetic ranges, variation in HbA1c may not drive cancer incidence [17].

DM also has a negative impact on cancer prognosis. This may be due to both biological factors, such as impaired immune surveillance and metabolic dysregulation, and clinical factors, such as delayed diagnosis, suboptimal cancer treatment, or contraindications to certain therapies. Moreover, the presence of diabetes can complicate the management of cancer by increasing the risk of treatment-related toxicities, surgical complications, and interactions between antidiabetic and anticancer drugs.

Accordingly, optimal glycemic control through individualized diabetes management is essential in these patients to improve cancer prognosis. Traditionally, long-term glucose control has been assessed via HbA1c; however, this marker does not capture acute glucose fluctuations or variability, which may be particularly relevant in patients undergoing cancer therapy. Recent advances, such as Continuous Glucose Monitoring (CGM), offer real-time insights into glucose dynamics and enable the evaluation of Time in Range (TIR): the percentage of time glucose remains within a target range (typically 70–180 mg/dL). These tools provide a more nuanced picture of glycemic control, allowing for precise management strategies that could positively influence cancer outcomes. This review explores the impact of glycemic control, including CGM-derived metrics, on cancer survival and discusses how integrating diabetes technologies into oncological care could improve prognoses in this growing patient population.

## Search strategy and selection criteria

A literature search was performed in the electronic databases PubMed, ISI Web of Science, and Scopus to identify relevant publications. Although a structured search strategy was applied, this review was not conducted as a formal systematic or scoping review and was not prospectively registered.

The search strategy combined controlled vocabulary terms and free-text keywords related to diabetes, glycemic control, and cancer. The following keywords and Medical Subject Headings (MeSH) were used in different combinations: “Diabetes Mellitus”, “type 2 diabetes”, “dysglycemia”, “hyperglycemia”, “glycemic variability”, “cancer”, “neoplasms”, “tumor progression”, “survival”, “prognosis”, “continuous glucose monitoring (CGM)”, “glucose monitoring”, and “anti-diabetic drugs”. These terms were combined using Boolean operators (AND/OR) with “cancer”, “neoplasms”, “oncologic outcomes”, “mortality”, “treatment tolerance”, and “immune checkpoint inhibitors”, as appropriate, to identify studies exploring the association between diabetes, glycemic control, and oncologic outcomes. The retrieved manuscripts were screened for additional relevant references.

We reviewed publications on the topic from 2000 to January 31, 2026, with particular attention to studies published within the last 10 years, including several relevant papers from 2020–2026, to ensure that the manuscript reflects the most recent evidence available. Only articles published in English in peer-reviewed journals were considered.

Studies focusing exclusively on pediatric populations, non-solid tumors, or those addressing only pharmacokinetic aspects without reporting oncologic or metabolic outcomes were excluded.

## Mechanisms linking hyperglycemia to cancer progression

**Fig. 1:**
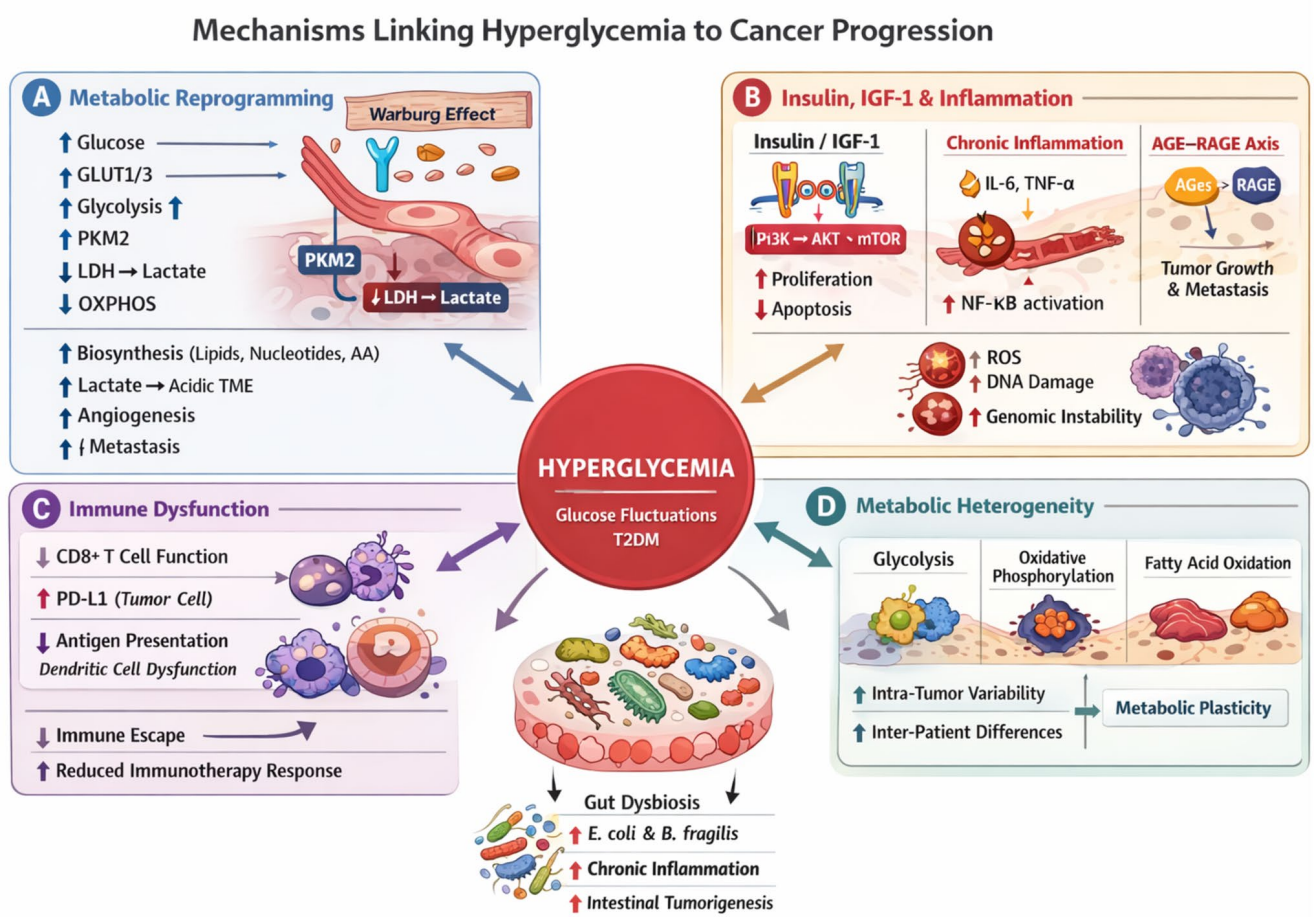
Mechanisms Linking Hyperglycemia to Cancer Progression. Cancer cells preferentially rely on aerobic glycolysis (Warburg effect), increasing glucose uptake and lactate production to sustain rapid proliferation. Hyperglycemia enhances glucose availability, fueling glycolytic flux and biosynthetic pathways. Overexpression of GLUTs, glycolytic enzymes (e.g., PKM2, LDH), and HIF-1α activation further support tumor survival, invasion, and angiogenesis. Excess glucose also promotes oxidative stress and genomic instability, linking dysglycemia to worse cancer outcomes, particularly in type 2 diabetes

Hyperglycemia drives cancer progression through multiple mechanisms, involving complex molecular and cellular pathways, which promote tumor growth and aggressiveness (Fig. 1).

### Metabolic effects: Warburg effect and glucose availability to tumor cells

Hyperglycemia may promote cancer progression primarily through metabolic reprogramming [18, 19]. Cancer cells characteristically exhibit increased glucose uptake and preferentially rely on aerobic glycolysis for energy production, a phenomenon known as the Warburg effect [19]. This metabolic shift involves the conversion of pyruvate into lactate rather than its entry into mitochondrial oxidative phosphorylation under aerobic conditions, thereby supporting rapid cell proliferation by supplying intermediates for nucleotide, lipid, and amino acid biosynthesis [18].

The Warburg effect is sustained by several mechanisms: i) overexpression of glucose transporters (GLUTs), which enhances glucose uptake [20]; ii) upregulation of glycolytic enzymes, such as lactate dehydrogenase and the M2 isoform of the glycolytic enzyme pyruvate kinase (PKM2), which redirects metabolism toward biosynthetic and antioxidant pathways, regenerating nicotinamide adenine dinucleotide (NAD), modifying the tumor microenvironment and promoting invasion, angiogenesis, and metastasis occurrence [18, 20]; and iii) activation of hypoxia-inducible factor-1α (HIF-1α), which further induces expression of glycolytic genes and supports survival in hypoxic tumor regions [18].

Persistent hyperglycemia leads to sustained systemic glucose availability, which may directly modify tumor metabolism and promote tumor growth, particularly in highly metabolically active cancers. High glucose concentrations can act as DNA-damaging agents, reducing tumor suppressor activity and inducing genomic instability [21]. In patients with T2DM, chronic hyperglycemia and glucose fluctuations have been correlated with worse cancer outcomes, likely due to enhanced metabolic support of tumor cells.

### Role of hyperinsulinemia, chronic inflammation, oxidative stress and microbiota

Hyperinsulinemia, commonly associated with T2DM and insulin resistance, plays a pivotal role in cancer progression. Elevated insulin levels stimulates both the insulin receptor and the IGF-1 receptor, pathways that are centrally involved in mitogenesis and inhibition of apoptosis [22]. These signaling cascades are frequently overexpressed in several cancer types, leading to increased proliferation and reduced cell death. Recent evidence suggests that specific microRNAs (miRNAs) regulating insulin signaling may contribute to the pathogenesis of both diabetes and cancer [23].

Furthermore, chronic low-grade inflammation associated with DM contributes to tumorigenesis through the production of inflammatory cytokines (IL-6, TNF-α), which can induce DNA damage, genetic instability, angiogenesis, and immune suppression [24]. Oxidative stress represents another critical link between elevated glucose levels and cancer. Hyperglycemia promotes the generation of reactive oxygen species (ROS), leading to oxidative DNA damage and genomic instability, both important contributors to cancer initiation and progression [25]. ROS can also cause damage to lipids, protein and DNA, and then induce carcinogenesis [7]. Advanced glycation end products (AGEs) further amplify oxidative stress and inflammation by binding to their receptors (RAGE) on cell surfaces. In cancer, AGEs promote tumor growth, proliferation, and metastasis through activation of inflammatory signaling pathways, including NF-κB and PI3K/Akt, making the AGE–RAGE axis a potential therapeutic target, with RAGE blockade emerging as a promising strategy [26].

Recent evidence also highlights the role of gut microbiota dysbiosis in cancer [27].The microbiota—defined as the community of microorganisms inhabiting specific human environments such as the gut—plays a fundamental role in digestion, metabolic regulation, and immune modulation [28]. Dysglycemia has been reported to impair microbiota composition thereby influencing systemic inflammation, nutrient absorption, and the tumor microenvironment, although this remains an area of active investigation [27]. In this context, Escherichia coli and Bacteroides fragilis have been associated with increased intestinal tumorigenesis in the setting of chronic inflammation [29].

### Impact of hyperglycemia on immune function

Glycemic dysregulation, mainly hyperglycemia, could have a significant effect on the immune system, through glycolysis, oxidative phosphorylation, AGEs-RAGE signaling and fatty acid oxidation, ultimately impairing antitumor immunity [30]. Dysregulated glucose availability and increased oxidative stress alter leukocyte function, reduce T-cell responsiveness, drive exhaustion-like phenotypes in CD8⁺ T cells, and modulate cytokine production. Hyperglycemia also impairs antigen presentation and alters dendritic cell maturation and macrophage polarization, thereby weakening immune surveillance and facilitating tumor immune escape [31]. Specifically, the tumor microenvironment becomes characterized by elevated glycolytic activity and nutrient depletion, which compromise immune cell metabolism and promote the expansion of immunosuppressive cell populations, resulting in a shift toward immune suppression [30]. Additionally, hyperglycemia has been shown to increase tumor cell expression of PD-L1 [32], a mechanism that may partially explain why T2DM represents an independent risk factor for poor prognosis in patients with advanced cancer receiving immunotherapy [33].

### Metabolic heterogeneity across tumor types

Different malignancies exhibit distinct metabolic dependencies, signaling pathways, and interactions with the tumor microenvironment, all of which can influence how dysglycemia affects tumor biology and treatment response. Tumor metabolism is highly dynamic and heterogeneous, varying not only across cancer types but also among patients and even within individual tumors during disease progression and exposure to therapy. Cancer cells undergo metabolic reprogramming to sustain proliferation, adapt to environmental stress, and maintain biosynthetic and energetic demands. However, these adaptations are not uniform: tumors may preferentially exploit different metabolic pathways depending on genetic alterations, tissue of origin, oxygen and nutrient availability, and treatments. In addition, metabolic plasticity enables cancer cells to shift between pathways in response to environmental constraints. This metabolic diversity contributes to variability in tumor growth, immune interactions, and sensitivity to anticancer treatments. Consequently, the biological and clinical impact of dysglycemia and altered glucose metabolism is likely to differ substantially across malignancies, underscoring the importance of considering tumor-specific metabolic contexts when interpreting the relationship between diabetes, glycemic control, and cancer outcomes [34, 35].

## The complex interplay between glycemic control and oncologic outcomes

Beyond its established role as a risk factor for cancer development, hyperglycemia has emerged as an independent prognostic factor in patients with cancers [11, 36]. Additionally, severe hypoglycemia and excessive glucose variability are independent predictors of worse prognosis and mortality in multiple types of cancers [23].

### Studies correlating hyperglycemia, hypoglycemia, HbAc1 levels, and cancer prognosis/mortality

Poor glycemic control, as reflected by elevated HbA1c levels, before and during cancer treatment has been associated with an increased risk of adverse outcomes, disease progression, and mortality [13, 37, 38].

During surgical procedures, interventional radiology, and systemic treatments, disturbances in glucose homeostasis—particularly hyperglycemia—represent significant risk factors for postoperative or treatment-related complications, including infection, hemorrhage, and delayed recovery [39]. Moreover, chronic hyperglycemia adversely affects tumor prognosis.

As summarized in Table 1, several studies have shown that cancer patients with DM tend to have worse outcomes compared to their non-diabetic counterparts, including higher cancer-specific and all-cause mortality, with an estimated 20–25% increase in mortality [11, 13]. Conversely, adequate glycemic control is associated with more favorable neoplastic outcomes, in terms of survival, progression and cancer recurrence [1, 40]. In T2DM, glucose fluctuations and chronic elevation of glucose levels correlate with worse cancer outcomes, likely due to enhanced metabolic support of tumor cells [11]. Indeed, cancer mortality among people with T2DM, is approximately 30–50% higher than in the general population, particularly for gastroenteropancreatic cancers [41]. A recent meta-analysis confirmed that, hyperglycemia is associated with worse overall (HR = 2.05, 95% CI 1.67–2.51; *P* < 0.001) and disease-free (HR = 1.98, 95% CI 1.20–3.27; *P* = 0.007) survival, compared to normoglycemia [42].

**Table 1:** Representative clinical studies linking dysglycemia and cancer prognosis/ mortality

Study	Study design / Population	Glycemic parameter	Cancer type	Main findings
Barua et al. [42]	Systematic review and meta-analysis /solid tumors	Hyperglycemia	Multiple	Hyperglycemia associated with worse OS (HR 2.05) and DFS (HR 1.98)
Seshasai et al. [43]	Pooled analysis of 97 retrospective studies/ *n* = 820,900 pts	Fasting glucose	Multiple	FG ≥ 126 mg/dL associated with increased cancer mortality (HR 1.39 vs 70–100 mg/dL)
Jee et al. [44]	Prospective cohort study/ *n* = 1,298,385 pts	Fasting glucose	Multiple	Linear increase in cancer mortality with rising FG (FG > 125 mg/dL associated with higher mortality vs FG < 90 mg/dL)
Ling et al. [41]	Population-based cohort study	Diabetes / hyperglycemia	Multiple	Higher cancer mortality in patients > 65 years
Renehan et al. [40]	Epidemiological analysis	Diabetes	Multiple	Diabetes associated with increased cancer-specific and all-cause mortality
Ling et al. / meta-analyses [11, 13]	Meta-analyses	Diabetes / glycemic control	Multiple	Relative increase in cancer mortality among patients with T2DM (30–50%) vs general population
Hope et al. [16]	Systematic review	HbA1c	Multiple	Higher HbA1c associated with increased cancer incidence in several tumors (CRC, pancreatic, lung, and gynecological)
Natalicchio et al. [38]	Narrative multidisciplinary review	HbA1c / glycemic control	Multiple	Poor glycemic control associated with worse oncologic outcomes (recurrence and mortality)

Abbreviations: *PTS* Patients; *FG* Fasting glucose; *HR* Hazard ratio; *vs* Versus; *CRC* Colorectal cancer

Age appears to modify this association. Ling et al. observed declining trends in cancer-specific mortality over time among patients under the age of 65 with cancer and T2DM, compared with those without diabetes. In contrast, mortality rates increased among those aged over 65, suggesting that a longer duration of exposure to hyperglycemia and elevated insulin levels may promote cancer cell growth and disease progression [41].

In a large analysis conducted on 820,900 subjects from 97 retrospective studies, it emerged that, as compared with the reference group (fasting glucose, FG, 70 to 100 mg/dL), patients with a FG of 126 mg/dL or more exhibit an HR of 1.39 (95% CI, 1.22 to 1.59) for cancer deaths. A HR for cancer death of 1.05 (95% CI 1.03–1.06) for every 1 mmol/L increase in glucose levels above 100 mg/dL was also reported [43]. Similarly, a ten-year prospective cohort study of 1,298,385 Koreans, reported a linear trend in cancer-related mortality with increasing FG in patients with a FG > 125 mg/dL compared with those with FG < 90 mg/dL, for most cancers [44]. However, glucose measurements were obtained at a single time point, which may not reflect persistent hyperglycemia.

A large a retrospective cohort study from Thailand assessed mortality risk in a population of adults with non-metastatic solid or hematologic malignancies, evaluating whether this risk differed by level and pattern of visit-to-visit glycemic control. The study included 1948 eligible patients (650 with diabetes and 1298 without), demonstrating a similar mortality risk between diabetic and non-diabetic patients. However, among those with diabetes, poor glycemic control, hypoglycemia, and high glycemic variability were associated with worse survival [45].

Several retrospective and observational studies have evaluated the impact of glycemic control on clinical outcomes across various cancers—including breast, colorectal, lung, pancreatic, and liver cancers—with most reporting a negative impact of poor glycemic control and an increased risk of recurrence and mortality, although a minority found no significant association [38]. Nevertheless, these studies are limited by their retrospective design, heterogeneous definitions of oncologic outcomes (OS, PFS, RFS), variability in glycemic markers (HbA1c vs. FG), inconsistent timing of measurements, and limited consideration of antidiabetic therapies. Despite these limitations, most of the evidence suggests that adequate glycemic control may favourably influence cancer outcomes, including survival, progression, recurrence, aggressiveness, and response to therapy.

Moreover, the relationship between glycemic control and outcomes, as well as treatment responsiveness, may vary substantially across malignancies, due to tumor heterogeneity, as above discussed [34, 35]. While several malignancies—such as breast, colorectal, pancreatic, and liver cancers—appear negatively influenced by diabetes or dysglycemia, other tumors, such as prostate cancer, show weaker or even inverse associations. A summary of representative evidence across major tumor types is reported in Table 2 [46–66]. It should be noted that most epidemiological studies assessing the relationship between metabolic disorders and cancer outcomes focus on the presence of diabetes rather than on specific indicators of glycemic control (e.g., HbA1c, fasting glucose, or glucose variability). Consequently, the available evidence largely reflects the impact of diabetes as a comorbidity, while the specific role of glycemic control per se remains less clearly defined and has been explored in a more limited number of studies.

**Table 2:** Diabetes, glycemic control, and prognosis across major tumor types: representative studies

Tumor type	Metabolic factor evaluated	Main association reported	Selected references
Breast cancer	Mostly presence of diabetes; few studies on HbA1c	Diabetes associated with worse overall and cancer-specific survival; possible benefit from metformin in diabetic patients	Hardefeldt et al. [46]; Lawrenson et al. [47]; Xiong et al. [48]; Rashmi et al. [49]
Lung cancer	Mainly diabetes status; few studies on pre-diabetes and HbA1C	Increased risk of lung cancer in diabetes and pre-diabetes. Some studies report poorer survival and increased treatment toxicity, though results are heterogeneous	Lu et al. [50]; Nguyen et al. [51]; Shen et al. [52]; Hua et al. [53]; Deng et al. [54]
Colorectal cancer	Mostly diabetes presence; limited data on glycemic control	Diabetes associated with higher incidence and worse prognosis in several cohorts	Mills et al. [55]; Zanders et al. [56]
Pancreatic cancer	Diabetes status (long-standing vs new-onset) – glucose control in non-diabetic populations	Long-standing diabetes increases risk; new-onset diabetes may represent an early manifestation of the tumor; optimal glucose control is associated with a lower risk of pancreatic cancer in non-diabetic populations	Ke et al. [57]; Feola et al. [58]; Zhang et al. [59]; Koo et al. [66]
Liver cancer (HCC)	Presence of diabetes	Diabetes associated with increased risk and worse outcomes, particularly in metabolic liver disease	Mantovani et al. [60]; Yang et al. [61]
Ovarian cancer	Diabetes status	Evidence heterogeneous; some studies suggest worse survival in diabetic patients	Slavchev et al. [62]
Prostate cancer	Presence of diabetes	Diabetes associated with lower incidence and unclear effect on cancer-specific mortality	Drab et al. [63]
Neuroendocrine tumors (NETs)	Presence of diabetes; some data on antidiabetic therapies	Diabetes may increase risk of certain NETs (particularly pancreatic NETs). Some retrospective studies suggest longer progression-free survival in NET patients treated with metformin	Pusceddu et al. [64]; Ruggeri et al. [65]

### Impact of glucose control on therapy adherence and efficacy

In the context of cancer therapy, poor glycemic control may negatively affect treatment efficacy and increase the risk of adverse events. Preclinical and clinical studies suggest that hyperglycemia can reduce antiproliferative effects of chemotherapy, by promoting proinflammatory milieu [21, 31, 67], particularly in the presence of diabetes-related complications such as nephropathy, vasculopathy, and neuropathy, and in patients receiving insulin therapy, which has been associated with increased cancer incidence and progression [68]. Conversely, more recent data from pancreatic ductal adenocarcinoma models indicate that a hyperglycemic state may enhance chemotherapy efficacy via suppression of glutathione biosynthesis (GCLC) and increased oxidative damage [69], highlighting tumor-specific metabolic interactions. This dichotomy underscores the context-dependent nature of hyperglycemia’s impact, influenced by tumor type, microenvironment, and drug class. Moreover, glycemic control at the initiation of chemotherapy may influence the risk of adverse events, infections, and hospitalizations during treatment, as well as the likelihood of chemotherapy dose reduction or discontinuation [70].

Hyperglycemia may also compromise the response to immune checkpoint inhibitors (ICIs), mainly in patients with advanced cancer [33, 71]. Retrospective studies in non-small cell lung cancer (NSCLC) provide the strongest evidence linking elevated baseline glucose or HbA1c levels with shorter progression-free and overall survival in patients treated with PD-1/PD-L1 inhibitors. Pre-existing diabetes has similarly been associated with reduced ICI efficacy and higher rates of early disease progression [33, 52]. Evidence also links dysglycemia to outcomes with targeted therapies. Patients with cancer and pre-existing diabetes exhibit higher mortality and recurrence rates following EGFR–tyrosine kinase inhibitor (TKI) treatment [72–74]. Additionally, patients with pre-existing DM and lung cancer harboring activating EGFR mutations have demonstrated poorer responses to first-line EGFR-TKI therapy [75]. Some studies suggest that hyperinsulinemia, rather than hyperglycemia alone, may contribute to resistance mechanisms in EGFR-TKI–treated NSCLC [76, 77]. The main findings from selected studies are summarized in Table 3 (preclinical studies) and Table 4 (clinical studies).

**Table 3:** Studies evaluating the impact of dysglycemia on cancer treatment efficacy and toxicity (preclinical models)

Study	Study design	Population / Cancer type	Glycemic parameter	Main findings
Gerards et al. [68]	Systematic review	Various cancers models	Hyperglycemia	Hyperglycemia may reduce chemotherapy efficacy and promote resistance mechanisms
Ma et al. [67]	Experimental study	Colon cancer cell lines	High glucose	Reduced antiproliferative effect of 5-fluorouracil
Vaziri-Gohar et al. [69]	Experimental study	Pancreatic ductal adenocarcinoma	Hyperglycemia	Increased chemotherapy sensitivity via glutathione suppression

**Table 4:** Representative studies evaluating the impact of dysglycemia on cancer treatment efficacy and toxicity (clinical models)

Study	Study design	Population / Cancer type	Glycemic parameter	Main findings
Hershey and Hession [70]	Prospective observational study	Patients with diabetes and cancer	Poor glycemic control	Association between poor baseline glycemic control and increased adverse events, infections, and chemotherapy dose reductions
Yekeduz et al. [71]	Retrospective study	Advanced cancers treated with ICIs	Chronic hyperglycemia	Hyperglycemia associated with reduced survival
Shen et al. [52]	Retrospective study	NSCLC patients treated with pembrolizumab	Baseline HbA1c and diabetes status	Association of higher HbA1c and diabetes with shorter PFS and OS
Yeo et al. [75]	Retrospective study	NSCLC with EGFR mutations	Diabetes / hyperinsulinemia	Diabetes associated with poorer response to EGFR-TKI
Ahn et al. [94]	Retrospective study	Breast cancer patients receiving adjuvant chemotherapy	Chemotherapy-related hyperglycemia	Association of hyperglycemia during chemotherapy with lower 5y RFS (82.3% vs 92.0%)
Brunello et al. [91]	Retrospective study	Patients receiving chemotherapy	Hyperglycemia	Association of hyperglycemia with increased chemotherapy-related toxicity
Mulla et al. [78]	Retrospective study	Cancer patients receiving ICIs	Treatment-related hyperglycemia	~ 9% developed hyperglycemia during therapy, often related to corticosteroid exposure

Abbreviations: *ICIs* Immune checkpoint inhibitors; *NSCLC* Non-small cells lung cancer; *PFS* Progression free survival; *OS* Overall survival; *EGFR* Epidermal growth factor; *EGFR-TKI* Epidermal growth factor tyrosine kinase inhibitors; *y* years; *RFS* Relapse free survival

Conversely, several anticancer therapies can directly or indirectly affect glucose metabolism, contributing to dysglycemia during treatment. The main mechanisms and metabolic effects associated with commonly used anticancer agents are summarized in Table 5. Observational cohorts indicate that approximately 9% of non-diabetic patients develop new-onset hyperglycemia during cancer treatment, largely driven by corticosteroid exposure (~ 76%), while immune-related diabetes accounts for approximately 10% of cases [78, 79]. Glucocorticoids—widely used for antiemesis, edema control, and premedication of chemotherapy or immunotherapy regimens – induce hyperglycemia by increasing hepatic gluconeogenesis, reducing peripheral glucose uptake, and promoting insulin resistance, leading to postprandial and circadian glucose excursions [80–83]. Even short-term exposure can precipitate clinically significant hyperglycemia, while chronic use increases the risk of diabetes and metabolic complications [80, 84, 85].

**Table 5:** Effects of anticancer therapies on glucose metabolism

Therapy class	Representative drugs	Mechanism affecting glucose metabolism	Metabolic effect	Key references
Glucocorticoids	Dexamethasone, prednisone, methylprednisolone	↑ Hepatic gluconeogenesis; ↑ insulin resistance; ↓ peripheral glucose uptake	Hyperglycemia, worsening glycemic control, steroid-induced diabetes	[80–85]
ICIs	Anti-PD-1 (nivolumab, pembrolizumab), anti-PD-L1 (atezolizumab), anti-CTLA-4 (ipilimumab)	Immune-mediated pancreatic β-cell destruction	Rare but severe insulin-dependent autoimmune diabetes (~ 0.2–1.9% incidence)	[86–89]
Combination ICIs	PD-1 + CTLA-4 inhibitors	Enhanced endocrine immune toxicity	Higher risk of immune-mediated diabetes compared with monotherapy	[86–89]
TKIs	Nilotinib, imatinib, sunitinib	Modulation of insulin signaling and glucose uptake pathways	Variable effects: hyperglycemia (e.g., nilotinib) or improved glycemic control/hypoglycemia (e.g., imatinib, sunitinib)	[90]
mTOR inhbitors	Everolimus, temsirolimus	Inhibition of insulin signaling and increased insulin resistance	Hyperglycemia and new-onset diabetes	[90]
Chemotherapy	Anthracyclines, taxanes, platinum agents	Metabolic stress, inflammation, steroid co-administration	Hyperglycemia, impaired glycemic control, increased treatment toxicity	[68, 90–93]

Abbreviations: *ICIs* Immune checkpoint inhibitors; *TKI* Tyrosine kinase inhibitors; *mTOR* Mechanistic target of rapamycin

True ICI-induced diabetes is rare overall (~ 0.2–1.9%) [86–88] but occurs mostly with PD-1/PD-L1 inhibitors and most commonly with PD-1/CTLA-4 combinations. By contrast, CTLA-4 monotherapy carries the lowest risk. Overall, ICIs can induce de novo DM in a low percentage of patients, sometimes requiring lifelong insulin replacement [89].

Targeted therapies, including tyrosine kinase inhibitors (TKIs) and the mTOR inhibitor everolimus, can exert heterogeneous effects on glucose metabolism, ranging from hyperglycemia—as observed with nilotinib and everolimus, due to increased insulin resistance—to hypoglycemia or improved glycemic control, as reported with imatinib or sunitinib, through their effects on glucose metabolism, insulin secretion or sensitivity, and glucose uptake [90]. Chemotherapy is also frequently associated with disturbances in glucose homeostasis, affecting both patients with pre-existing diabetes— with potential worsening of survival [42]—and those without prior metabolic disease. Hyperglycemia can reduce chemotherapy tolerance, contributing to toxicities such as neutropenia, nephropathy, vasculopathy and neuropathy, and may lead to treatment discontinuation [68, 70, 91, 92]. Incidence rates of chemotherapy-related hyperglycemia vary widely from 20 to 60% depending on population, regimen, and use of concomitant drugs [93]. In a Korean cohort of breast cancer patients, transient hyperglycemia during adjuvant chemotherapy occurred in approximately 20% of cases and was independently associated with relapse-free survival (RFS) with a 5-year RFS rates of 92.0% and 82.3% in the euglycemia and hyperglycemia groups, respectively (*p* = 0.011) [94].

Nevertheless, the current literature is limited by heterogeneous definitions of hyperglycemia, the predominance of retrospective design of most studies, and confounding from concomitant drug administration, particularly corticosteroids.

Overall, these findings suggest that glycemic control is a crucial but modifiable determinant of cancer treatment benefit, supporting the need for integration of DM management into oncologic care to optimize both metabolic and cancer-related outcomes [95].

### Potential antitumor effects of antidiabetic drugs

In the context of the complex relationship between diabetes and cancer, it is important to briefly acknowledge the potential antitumor effects of some antidiabetic therapies, including metformin, glucagon-like peptide-1 receptor agonists (GLP-1RAs), and sodium–glucose cotransporter-2 inhibitors (SGLT2i). Beyond their metabolic actions, several glucose-lowering agents may influence pathways involved in cell proliferation, apoptosis, and tumor metabolism [96].

Among these, metformin has attracted particular attention for its potential impact on cancer incidence and outcomes, although clinical evidence remains heterogeneous. By improving insulin sensitivity and lowering circulating insulin and IGF-1 levels, metformin may attenuate mitogenic signaling, while direct cellular effects include activation of AMP-activated protein kinase (AMPK) and inhibition of the mTOR pathway, leading to metabolic stress and reduced tumor proliferation [97]. Observational studies in patients with type 2 diabetes have reported a lower incidence of several malignancies, including colorectal and pancreatic cancers, among metformin users compared with other antidiabetic therapies [98, 99]. In breast cancer, epidemiological data suggest reduced risk and improved survival, particularly among diabetic patients [100]. Similarly, retrospective analyses have reported longer progression-free survival in metformin-treated patients with pancreatic neuroendocrine tumors [64], although prospective confirmation is still required.

Other antidiabetic drug classes have also been explored for potential anticancer properties. GLP-1 receptor agonists have demonstrated antiproliferative and pro-apoptotic effects in several experimental models, partly through modulation of PI3K/AKT/mTOR and cAMP signaling pathways and through indirect metabolic effects such as improved insulin sensitivity and reduced hyperinsulinemia [65, 101, 102]. Similarly, SGLT2 inhibitors may limit tumor growth by reducing glucose availability and interfering with glycolytic metabolism and AMPK- and mTOR-related signaling pathways [65, 103]. Observational clinical data also suggested a possible protective association. A recent retrospective cohort study reported a lower overall tumor incidence among patients with type 2 diabetes treated with SGLT2 inhibitors, with reductions particularly observed for lung and ovarian cancers [104]. However, larger meta-analyses of randomized trials have not demonstrated a significant change in overall cancer risk with SGLT2 inhibitor therapy [105]. Therefore, the clinical relevance of these potential anticancer effects remains uncertain and warrants further investigation in prospective studies.

## Advances in Glucose Monitoring: CGM and Time in Range (TIR) as advanced metrics.

Over the past several years, self-monitoring of blood glucose (SMBG) has been complemented in glycemic monitoring by continuous glucose monitoring (CGM). CGM involves the subcutaneous insertion of a sensor that automatically measures interstitial glucose levels continuously, providing near–real-time glucose data [106]. This approach can address several limitations of SMBG, including the difficulty of detecting unrecognized hypoglycemia, the lack of predictive systems for hypo/hyperglycemia, and potential measurement errors related to hand contamination [107]. Several parameters can be derived from sensor data**;** among the most commonly used and clinically relevant there are time in range (TIR), time above range (TAR), and time below range (TBR), as well as coefficient of variation (CV), glucose management indicator (GMI), and mean glucose[106]. Key CGM-derived metrics and their potential relevance in oncology patients are summarized in Table 6.

**Table 6:** Continuous glucose monitoring metrics and their potential relevance for metabolic management in oncology patients

CGM metric	Definition	Clinical relevance in diabetes care	Potential implications in oncology patients
Time in Range (TIR)	Percentage of time glucose levels remain within the target range (typically 70–180 mg/dL)	Key indicator of overall glycemic control and predictor of diabetes complications	May help optimize metabolic control during cancer therapy, potentially improving treatment tolerance and outcomes
Time Above Range (TAR)	Percentage of time glucose levels exceed the target range (> 180 mg/dL)	Reflects hyperglycemia burden and risk of complications	Persistent hyperglycemia may promote tumor progression, impair immune function, and reduce treatment efficacy
Time Below Range (TBR)	Percentage of time glucose levels fall below the target range (< 70 mg/dL)	Indicates risk of hypoglycemia	Hypoglycemia may increase morbidity, hospitalization risk, and negatively impact cancer prognosis
Coefficient of glucose variability (GV)	A measure of dynamic glucose variability expressed as percentage coefficient of variation and calculated as 100 × (SD divided by mean glucose); coefficient of variation is correlated with time below range	High variability associated with oxidative stress and vascular complications	May worsen systemic inflammation and metabolic stress during cancer treatment
Avarage glucose level (AG)	mean blood sugar level calculated from continuous readings over a period (like 14 days)	Indicator of overall glycemic exposure	Persistent hyperglycemia may support tumor metabolism and affect survival
HbA1C	Automatic estimation of glycated hemoglobin levels based on sensor-detected blood glucose levels	Standard marker of long-term glycemic control	Elevated HbA1c has been associated with increased cancer risk, poorer prognosis, and higher mortality in several malignancies; however, its reliability may be reduced in cancer patients due to anemia, transfusions, or chemotherapy-induced changes in erythrocyte turnover

Multiple studies and meta-analyses have shown that CGM use—when coupled with appropriate interpretation and therapeutic adjustment—is associated with improved glycemic control in diabetic patients. CGM also enables a more accurate characterization of an individual’s glycemic profile, highlighting abnormalities that may not be evident when relying solely on HbA1c, such as marked glycemic variability or frequent (including asymptomatic) hypoglycemia [108–110].

It is now well established that improvements in CGM metrics are associated with better outcomes across several conditions. Notably, such improvements have been shown to correlate with reduced risk of certain chronic diabetic complications, such as retinopathy [111] and foot ulcers [112]. In addition, accumulating evidence associates improved CGM metrics with lower cardiovascular risk, including reductions in cardiac ischemia, stroke, heart failure, and hospitalization rates [113, 114], although some studies remain inconclusive, particularly regarding the progression of atherosclerotic damage [115].Regarding non-diabetes-related diseases, the available evidence is more limited.

Managing diabetes in cancer patients is particularly challenging for physicians, as poor glycemic control is associated with worse cancer survival, increased risk of comorbidities, and mortality. In addition, a cancer diagnosis is associated with reduced medication adherence, particularly in advanced disease [52, 116].Furthermore, a recent survey among American oncologist, highlighted substantial variability in hyperglycemia management during chemotherapy [16]. In this setting, oncologists often rely on HbA1c, even though HbA1c may be altered by common conditions such as anemia, transfusion exposure, and impaired hematopoiesis. In such circumstances, CGM may provide a more reliable depiction of glycemic patterns and facilitate earlier detection of hypoglycemia [93, 117].

Although direct evidence linking CGM use to improved cancer outcomes remains limited with the available studies constrained by small sample sizes, heterogeneous tumor types, and diverse clinical endpoints, it is reasonable to infer that the adoption of these technologies may contribute to better prognosis in cancer patients through multiple pathways. Indeed, improved glycemic monitoring may indirectly influence cancer outcomes by enhancing treatment adherence, reducing metabolic complications, enabling optimal dose intensity of anticancer therapies, and potentially modulating immune and inflammatory pathways associated with dysglycemia. Among the available evidence, it is noteworthy that hypoglycemia is associated with a poorer prognosis and several studies indicate that CGM use—particularly when combined with early specialist input from a diabetologist—can reduce hypoglycemic events, especially in frail patients undergoing systemic therapy [118]. In particular, the prospective Oncodiab trial implemented a multidisciplinary follow-up protocol in which CGM was applied during the first two cycles of chemotherapy. The coordinated intervention of diabetologists, pharmacists and oncologists resulted in improved glycemic control and more effective optimization of glucose-lowering therapy [119].

GCM has proven useful also in detecting hyperglycemia in patients undergoing cancer treatment (particularly steroid-induced hyperglycemia) [120] and in monitoring blood glucose levels in patients in critical care settings such as intensive hematology care, where it can also help ensure a safe treatment choice [121]. Moreover, CGMs have proven useful in the post-operative management of certain neoplasms. For example, CGM can improve assessment and characterization of dumping syndrome after distal or total gastrectomy [122, 123]. In patients undergoing colorectal cancer resection or those requiring enteral nutrition for esophageal cancer, CGM use has been associated with more favorable clinical outcomes, including accelerated wound healing, reduced incidence of post-operative complications and shorter hospital stay [124, 125]. Specifically, the study by Wang et al., conducted on 181 patients with Type 2 Diabetes undergoing colorectal cancer surgery, highlighted that patients managed postoperatively with CGM had better clinical outcomes, improved anastomotic healing, and shorter hospital stays compared to those monitored with capillary blood glucose. These benefits are attributed to technology’s ability to provide real-time glycemic data, as well as early detection of hyperglycemia and nocturnal hypoglycemia, thereby providing clinicians with more informed data which leads to timely intervention and improved glycemic stability [95].

However, evidence linking CGM-derived metrics directly to cancer outcomes or treatment response remains limited. In a prospective cohort study of 6,225 individuals with type 2 diabetes, lower TIR was inversely associated with both total cancer mortality and hepatocellular cancer mortality; notably, patients with TIR ≤ 70% had the highest risk of cancer mortality within 5 years [126]. While these findings suggest that TIR may have potential prognostic relevance, causality cannot be inferred and residual confounding has remained likely.

It is important to underline that, given the significant positive impact of CGM on the Quality of Life (QOL) of diabetic patients, although disease-specific data for cancer are not yet fully available, QOL remains a primary goal in cancer management. The strong consensus regarding the expanded use of CGM in clinical practice including inpatient settings, as supported by the latest ADA guidelines, will undoubtedly provide more comprehensive evidence in the future regarding the benefits of these technologies for the oncological patient journey.

Finally, given its benefits, the latest American Diabetes Association guidelines recommend CGM use at the onset of diabetes and at any time thereafter for diabetic patients of any age on insulin therapy, and those on non-insulin therapy with a hypoglycemia risk, or whenever the technology aids in disease management [127].In addition, it is encouraged to maintain CGM use even during hospitalization if requested by the patient and clinically appropriate (C). In the future, this will provide access to an increasing volume of data that may also be utilized to evaluate oncological outcomes in patients using CGM.

Larger, multicenter prospective studies specifically designed to evaluate CGM metrics in oncology populations are therefore needed to clarify whether improving time-in-range and reducing glycemic variability can translate into better cancer outcomes or treatment responses.

## Clinical management and challenges

Glycemic management in patients with coexisting diabetes and cancer is complex and challenging, and these patients should be offered a multidisciplinary approach consisting of cancer and diabetes specialists working collaboratively to optimize care.

Over time, the therapeutic armamentarium against cancer has expanded substantially, and multiple classes of anticancer drugs can induce hyperglycemia, either worsening pre-existing diabetes or precipitating new-onset diabetes. Cancer therapies—including corticosteroids, selected chemotherapies, immune checkpoint inhibitors, tyrosine kinase inhibitors, mTOR inhibitors, and somatostatin analogues—can affect glucose homeostasis through distinct mechanisms, increasing the risk of dysglycemia and complicating metabolic management [128, 129]. Furthermore, nausea, vomiting and anorexia secondary to oncology treatments can further destabilize glycemic control, particularly in patients receiving glucose-lowering agents, due to an increased risk of hypoglycemia [118].

In patients with diabetes and cancer, clinicians may understandably prioritize oncologic management and accept less stringent glycemic control as an anticipated adverse consequence of treatment [39, 130, 131]. However, adherence to glucose-lowering therapy often declines after a cancer diagnosis, particularly among patients with poorer prognosis or advanced TNM stage [116]. Similarly, self-management behaviours such as intermittent capillary blood glucose determination (finger pricks) may decreased during cancer care [38], and the frequency of medical visits for diabetes management is lower than in the pre-cancer period [132].

Although achieving glycemic control during cancer therapy is challenging, therapeutic nihilism should be avoided, as diabetes and cancer can exacerbate each other’s morbidity. Patients with diabetes and cancer appear to be at increased risk of preventable acute diabetes complications after cancer diagnosis, with cumulative rates increasing 1.5- to twofold within the first year [132]. The reciprocal burden of both diseases is amplified by the fact that many affected individuals are aged ≥ 65 years and frequently present with renal dysfunction, cardiovascular comorbidities, and polypharmacy.

Therefore, diabetes management in patients with cancer should be improved through a personalized approach that balances glycemic control with oncologic priorities and individual prognosis. As emphasized by Chowdhury and Jacob, clinicians and patients must also consider long-term survivorship (“living with and beyond cancer”), as prevention of diabetes-related complications remains clinically relevant in many individuals who will live for years after diagnosis [39].

### Personalizing glycemic targets in cancer patients

European and American diabetes guidelines emphasize individualized glycemic targets based on age, comorbidities, and willingness and ability to engage in self-management [133]. Moreover, updated recommendations include specific guidance for glycemic management during cancer treatment and immunotherapy [134, 135].Accordingly, in patients with diabetes and cancer, glycemic goals should be tailored to prognosis, ongoing therapies, clinical status, comorbidities (e.g., renal, cardiac, or hepatic dysfunction), life expectancy, and patient preferences. In a patient with favourable prognosis, stricter targets may be appropriate to reduce acute complications of cancer therapy (e.g., surgical infections) and prevent long-term diabetes complications. Conversely, in patients with limited life expectancy or high vulnerability, strict targets may offer limited benefit and increase the risk of hypoglycemia; therefore, less stringent goals may be appropriate to preserve quality of life. Overall, clinicians should engage patients in careful discussion of the risks and benefits of glycemic targets in the context of cancer trajectory and treatment burden.

### Preventing hypoglycemia and managing hyperglycemia

Glycemic fluctuations are common in cancer patients due to chemotherapy, corticosteroid use, anorexia, nausea and vomiting or impaired liver and kidney function. Hypoglycemia, particularly in patients receiving insulin or sulfonylureas, can be dangerous and should be prevented through careful adjustment of therapy [23]. Recurrent and severe hypoglycemia is associated with increased morbidity, mortality, prolonged hospitalization, readmission, and healthcare costs [136, 137] and has been also associated with a worse oncological prognosis [118]. Hyperglycemia, conversely, can impair cancer outcomes by reducing treatment efficacy, increasing infection risk, delaying wound healing, [68, 138–140], and contributing to anticancer drug dose reduction, delay, or discontinuation [72, 91, 141]. Poor glycemic control is also associated with greater pain severity and fatigue [39]. In long-term cancer survivors, persistent hyperglycemia increases the risk of microvascular and macrovascular complications [142, 143].

Therefore, glucose should be actively managed in patients with diabetes and cancer. Clinicians should recognize therapies that predispose to hyperglycemia and implement proactive strategies. Screening for undiagnosed diabetes before initiation of high-risk therapies is essential. Management of glucocorticoid-induced hyperglycemia—preferably with early involvement of the diabetes team—may reduce large glucose excursions. Patients should be supported in developing individualized plans for cyclical regimens to anticipate predictable glycemic changes. Patients with type 1 diabetes or those requiring enteral feeding require close specialist follow-up [39].

### Use of CGM to personalize diabetes management during cancer treatment

CGM, is a safe and well-tolerated tool, that allow more accurate, real-time assessment of glycemic profiles and could be particularly valuable in cancer patientsby enabling timely therapeutic adjustment, supporting early intervention for significant fluctuations, and reducing the risk of acute complications [89]. CGM can support individualized glucose management and reduce high-risk variability by providing a comprehensive 24-h glucose profile and identifying nocturnal or asymptomatic hypoglycemia. This approach may improve glycemic stability and enhance self-management capacity, quality of life, and treatment satisfaction.

To date, relatively few studies have evaluated CGM specifically in patients with diabetes and cancer. [Preliminary evidence suggests that CGM may reduce hypoglycemia and improve TIR during chemotherapy [118–120, 144], in postoperative setting [125] and in patients receiving enteral nutrition [124].

However, no formal guidelines currently define CGM indications specifically for oncology populations, and use remains individualized. Cost represents a major barrier to widespread implementation, and future cost-effectiveness analyses should stratify patients by diabetes severity and cancer trajectory to identify subgroups most likely to benefit. Potential high-yield candidates include patients receiving frequent corticosteroids, those with unrecognized hypoglycemia, high glycemic variability, or enteral feeding requirements. Finally, patient and caregiver education is essential to ensure appropriate interpretation of CGM trends and responses to alarms, alongside frequent data review—particularly during the first weeks of oncologic treatment—and coordinated multidisciplinary care involving diabetologists, oncologists, nurses, and dietitians.

**Fig. 2:**
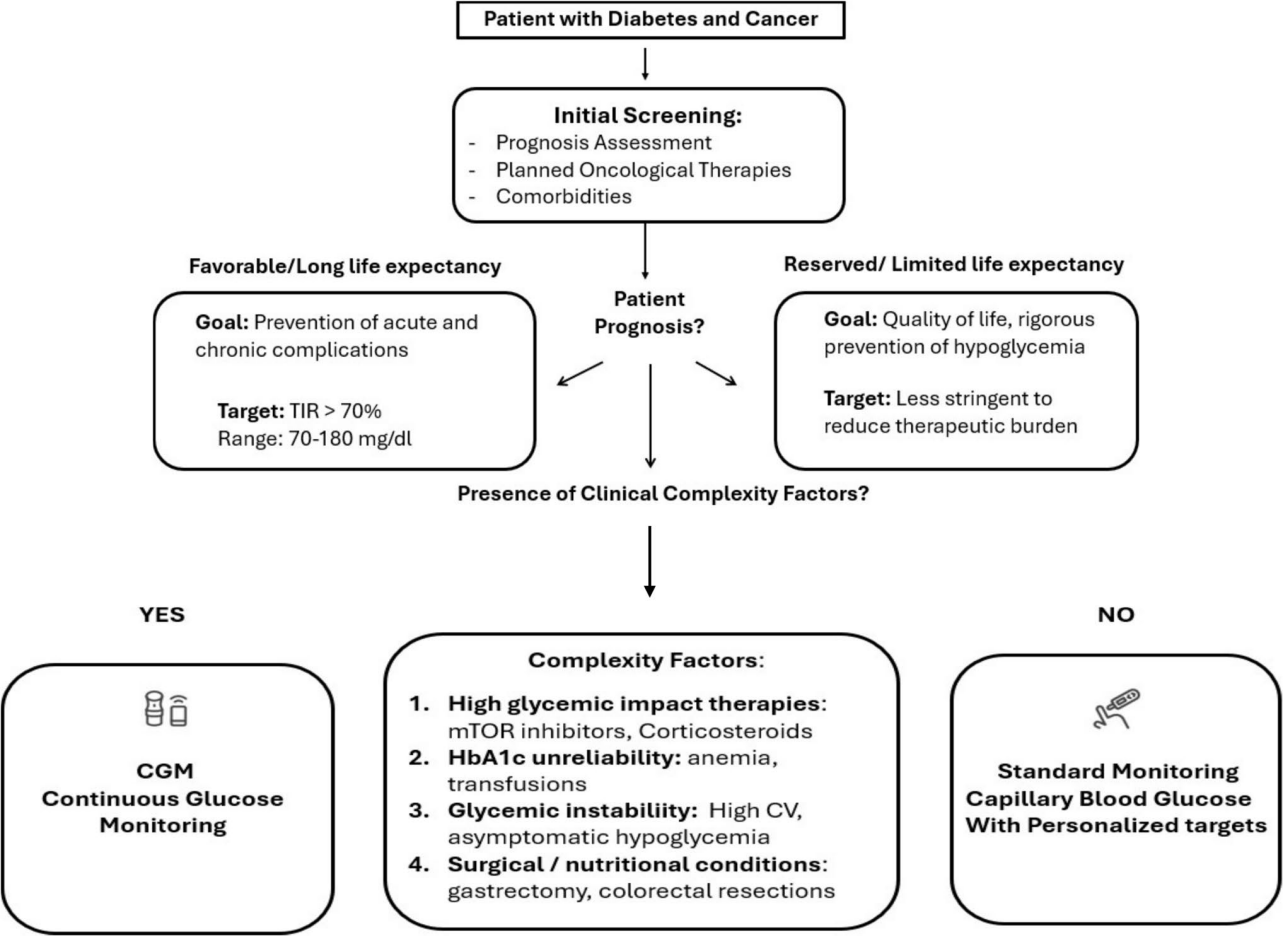
Proposal for a personalize diabetes management in cancer patients. An integrated, multidisciplinary approach combining oncology and diabetology is proposed to optimize glycemic control during cancer therapy. Continuous glucose monitoring enables real-time assessment of glucose variability and hypoglycemia risk, allowing timely therapeutic adjustments. Personalized metabolic management may reduce treatment-related complications, improve therapy tolerance, and support better oncologic outcomes

An integrated, multidisciplinary approach combining oncology and diabetology is proposed to optimize glycemic control during cancer therapy (Fig. 2).

## Limitations of current evidence

Several limitations of the current evidence should be acknowledged. Most studies evaluating the relationship between dysglycemia and cancer outcomes are observational and frequently retrospective, which limits the ability to infer causality. Therefore, the reported relationship between poor glycemic control and adverse oncologic outcomes should be interpreted cautiously, and a clear distinction between association and causation must be maintained. Another important limitation relates to the substantial tumor-specific and metabolic heterogeneity across cancer types, as above discussed. Consequently, the influence of hyperglycemia or glycemic variability on cancer outcomes is unlikely to be uniform across tumor types, and extrapolation of findings from one cancer setting to another should therefore be interpreted with caution. Residual confounding remains likely, as patients with diabetes often differ from non-diabetic individuals with respect to age, obesity, comorbidities, lifestyle factors, and exposure to medications that may independently influence cancer prognosis. In addition, reverse causality cannot be excluded, since advanced cancer, systemic inflammation, or cancer-related treatments may alter glucose metabolism and glycemic parameters. The available literature is also heterogeneous regarding tumor types, glycemic markers (e.g., HbA1c, fasting glucose, or CGM-derived metrics), timing of measurements, and definitions of oncologic outcomes. These methodological differences complicate direct comparisons across studies and limit the generalizability of findings. Therefore, while the available evidence supports a consistent association between dysglycemia and adverse oncologic outcomes, prospective and well-controlled studies are required to clarify the causal nature and clinical magnitude of this relationship.

## Conclusions and future directions

**Fig. 3:**
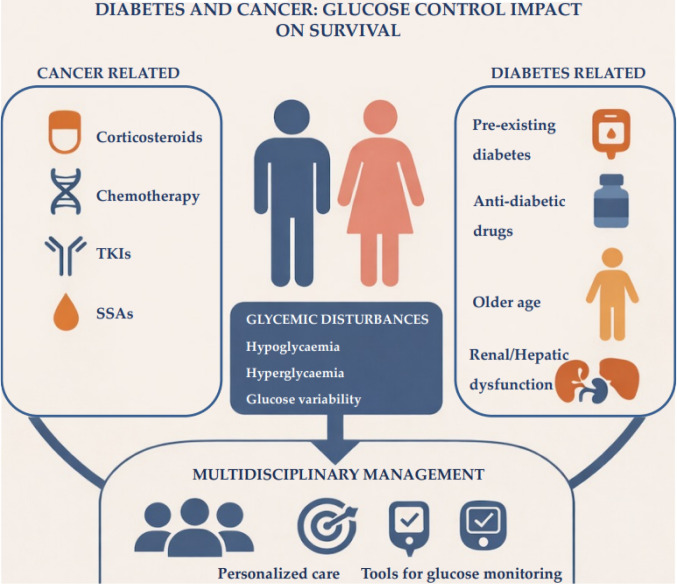
Multidisciplinary and Personalized Glycemic Management in Patients with Diabetes and Cancer. This figure illustrates the bidirectional and dynamic interplay between cancer, anticancer therapies, and glucose homeostasis, emphasizing the need for a multidisciplinary and personalized approach to glycemic management

In recent years, there has been increasing research interest in the complex relationship between dysglycemia, diabetes, glycemic control, and cancer outcomes, highlighting the roles of glycemic variability, metabolic reprogramming, and the potential impact of antidiabetic therapies on tumor biology.The evidence summarized in this review highlights the relevance of dysglycemia as important modifiers of cancer risk, progression, treatment tolerance, and survival (Fig. 3). Hyperglycemia, glucose variability, and hyperinsulinemia act through intertwined metabolic, inflammatory, and immune pathways that promote tumor growth and impair antitumor responses, while also increasing treatment-related complications. Emerging evidence suggests that acute hyperglycemia, hypoglycemia, and glycemic variability are associated with worse outcomes among cancer patients with diabetes compared to their non-diabetic counterparts. Optimizing glycemic control may therefore contribute to improved cancer outcomes. Although HbA1c remains a cornerstone of glycemic assessment, it incompletely captures the dynamic glucose disturbances commonly observed during cancer therapy.

Advances in CGM and metrics such as TIR provide a more comprehensive and clinically meaningful assessment of glycemic control and represent promising tools to personalize diabetes management in oncology settings. Preliminary studies indicate that CGM-guided interventions can reduce hypoglycemia, improve glycemic stability, and potentially support better adherence and tolerance to cancer treatments. However, the current evidence base remains largely observational, heterogeneous, and frequently confounded by disease severity, corticosteroid exposure, nutritional changes, and treatment selection, limiting causal inference and generalizability. Robust prospective evidence linking CGM-derived metrics to hard oncologic endpoints (e.g., survival, recurrence, and response) is still lacking.

Future research should prioritize well-designed prospective trials to define optimal glycemic targets across cancer types, stages, and therapies, and to clarify whether improving CGM metrics translates into meaningful improvements in treatment efficacy, safety, and survival. Integrating diabetology and oncology care through multidisciplinary models, supported by digital health technologies, will be essential. Ultimately, recognizing glycemic control as a modifiable factor in cancer care may provide new opportunities to improve metabolic management and enhance the overall quality and effectiveness of oncologic treatment in this vulnerable population.

## Data Availability

No datasets were generated or analysed during the current study.
